# Friction and Wear Mechanism Analysis of Polymer Flexible Cable Using a High Natural Frequency Piezoelectric Sensor

**DOI:** 10.3390/s20041044

**Published:** 2020-02-14

**Authors:** Jing Ni, Xu Ren, Junqiang Zheng

**Affiliations:** School of Mechanical Engineering, Hangzhou Dianzi University, Hangzhou 310018, China; nj2000@hdu.edu.cn (J.N.); renxu@hdu.edu.cn (X.R.)

**Keywords:** cable reliability, wear mechanism, friction and wear test, flexible cable

## Abstract

The friction and wear of flexible cables are the main factors that cause electrical breakdown and insulation aging, and they greatly reduce the reliability and safety of robots. In order to enhance the reliability and safety of the robot, it is of great necessity to investigate the friction and wear mechanisms of the flexible cable. In this research, the friction and wear mechanisms have been discussed. The effects of relative speed, ambient temperature, and positive pressure on the flexible cables are considered by an orthogonal frictional movement. The cable friction force has been measured by a piezoelectric sensor with high natural frequency characteristics. The relations among friction and different factors affecting friction have also been discussed. The results show that the relative speed and the ambient temperature are the main factors affecting the friction and wear of the cable; the main form of flexible cable wear is mechanical-force chemical friction and wear. Those discoveries will greatly deepen the understanding of the friction and wear mechanisms of flexible cables, and will be beneficial to robot cable-reliability design.

## 1. Introduction

Industrial robots can replace manual work in high-intensity environments such as high temperature and high pressure. In particular, robots can also replace people to perform repetitive tasks, which is more efficient than using humans, and this can become an essential part of industrial production [1,2]. As the key components in robot systems, the flexible cables used in robots are the main carriers for transporting the control signals and power [3]. The stability of the flexible cable directly determines the safety of a robot. The following problem is that the drag and friction in the cable can pose difficulties in working conditions [4]. The robot flexible cable follows the robot frequently, which causes severe insulation aging and relative sliding between two cables. The severe insulation wear directly causes the flexible cable failure [5]. In addition, the wear of the flexible cable insulation layer indirectly exacerbates the aging of the cable and reduces the cable service life, which drastically reduces the safety and reliability of the robot. Currently, the friction and wear mechanisms of the flexible cable are not clear, and the assessment of the reliability of the flexible cable is not complete—these seriously hinder the development of industrial automation. Therefore, studying the friction and wear characteristics of a robot flexible cable under different working conditions is of great importance to extend the service life of a flexible cable and improve the reliability of a robotic system.

In the past decades, many scholars have studied the characteristics of cables. In order to study the friction and wear characteristics between Cu-Ag alloy wires and pure copper conductors, Hu Y et al. carried out several experimental tests. The effects of current and voltage on the friction between Cu-Ag-silver contact wires and pure copper contact wires were tested at high speed motion. Finally, they have found that friction and wear are the main factors in contact-wire failure [6]. It has shown the importance of friction and wear in contact wires. Songhan Z. et al. studied the friction and wear damage of the cables under local bending behavior, based on the vibration damage identification technology [7]. When people realized the importance of friction and wear on rigid cables (rigid cables made entirely of metal conductors, such as transmission cables, contact wire). In order to effectively control friction and wear, scholars have researched what factors affect the friction and wear of cables. Yang H.J. et al. studied the friction and wear properties of a contact strip rubbing against a contact wire. The result showed that the interfaced relative speed and the surface temperature have a certain effect on current and wear [8]. Jia S.G. has studied the sliding wear behavior of the Cu-Ag-Cr wire and found the effects of positive pressure and sliding speed on the wear of Cu-Ag-Cr wire. The abrasive wear and arc corrosion are the main mechanisms of wear during electrical sliding [9]. The study of cable friction and wear is not only of scientific significance but also of great engineering significance. It can provide great data support for the design of cable reliability. For example, Ismailov G. M. proposed a new method to determine the friction force of cable, in which the cable parameters are taken into account. The strength of the cable is determined by the mechanical stress in the cable contact element, which provides reference data for the cable design [10]. The research results of these scholars provide a great reference for the selection of working conditions in this study. However, the above research focuses on rigid cables like transmission cables, contact wire, and so on, not a flexible cable. Nowadays, the research on the friction and wear of flexible cables widely used in various industries is insufficient.

With the development of the electrical industry, flexible cables make up half of the number of cables used in the electrical industry. More and more scholars pay attention to the research on characteristics related to a flexible cable. To this end, Perier V. et al. studied bridge cables under the fretting friction, and only the effects of the corrosive environment were considered. They put the surface intact cable in a corrosive solution and performed a fretting friction and wear test of the cable. The result shows that lubrication and galvanizing can improve the anti-fretting fatigue performance of electric wires [11]. Coincidentally, Kotov M. A. et al. studied how a cable base’s damage reduces the belt strength in the operation of rubber cable conveyor belts under the cable with an undamaged surface. Finally, they set up the standards of admissible wear and tear, which are essential for reducing hazards and increasing belt service life [12]. On the safe use of cables, Chang F. also did some research. In his work, the Hifax cable samples must have no cracks or abrasions on the surface, and the DC resistivity and AC breakdown strength of flexible polypropylene cable insulation materials have been measured at selected temperatures. He has concluded that the electrical stress coefficient of resistivity of Hifax cable insulation increases with temperature. Charge trapping and de-trapping processes are present in these cable insulations [13]. These results show that the safe use of flexible cables is most strongly affected by the chemical factors of the working conditions of the insulation layer. In order to study the effect of chemical wastewater on cables, Anwar Ul-Hamid et al. discussed the corrosion failure mechanism of cables in polluted environments. They buried intact cables in soil containing different wastewaters and studied their corrosion and aging phenomena. This information is useful for planting engineers and project managers working in an industry that uses chemicals [14]. In the above work, the dielectric properties of flexible insulated cables have been studied through various aspects to ensure the safe use of cables. However, most of them have been studied on cables with complete surfaces, the sample of those studies have required no damage and wear scars on the cable surfaces. They ignore the effects of friction and wear, and there are some errors with the actual cable situation. This results in inaccurate experimental results and some errors.

From the above, the researches on cables are focused on two main aspects. On the one hand, the previous work mainly focused on the tribological properties and practical applications between rigid cables. Rigid cables are mainly formed by winding metal wires, and their surface is often regarded as a rigid body that does not deform. On the other hand, scholars pay more attention to the reliability of flexible insulated cables, which mainly manifests research on the effects of aging of the complete insulation layer on its performance under different chemical and temperature corrosion. For those researches, the samples required no damage and no scars. They ignored the effects of friction and wear on the reliability of flexible cables. However, the friction and wear of the flexible cable greatly accelerated the aging of the insulation layer. It is necessary to study the friction and wear performances of flexible cables. Although much research has been done on the friction and wear of rigid cables, the existing research on friction and wear of rigid cables cannot replace specific research on the friction and wear of flexible cables. Because the insulation layer of a flexible cable is composed of an elastomeric polymer material, its friction is greatly different from those of a rigid cable. For the reliable and safe use of flexible cables, an in-depth understanding of the friction and wear mechanisms of flexible cables is expected. At present, there is an urgent need to study the friction and wear of the flexible insulated cable.

The purpose of this study is to research the effects of relative speed, ambient temperature, and positive pressure on the friction and wear of flexible cables. The cable’s insulation layer is made of polyvinyl chloride-based polymer. A flexible cable friction and wear test system designed to carry orthogonal friction experiments, and the friction forces of the cables have been recorded by the piezoelectric sensor [15,16,17]. The surface and chips of the wear obtained from the test were analyzed by a microscope. The flexible cable-wear mechanism has been built based on the analysis of results, and the influence of three factors on cable friction are summarized. In addition, the research results will provide basic data for the design of robots, and help with the safe use of robot flexible cables.

## 2. Experimental Details

### 2.1. Experiment System

**Figure 1:**
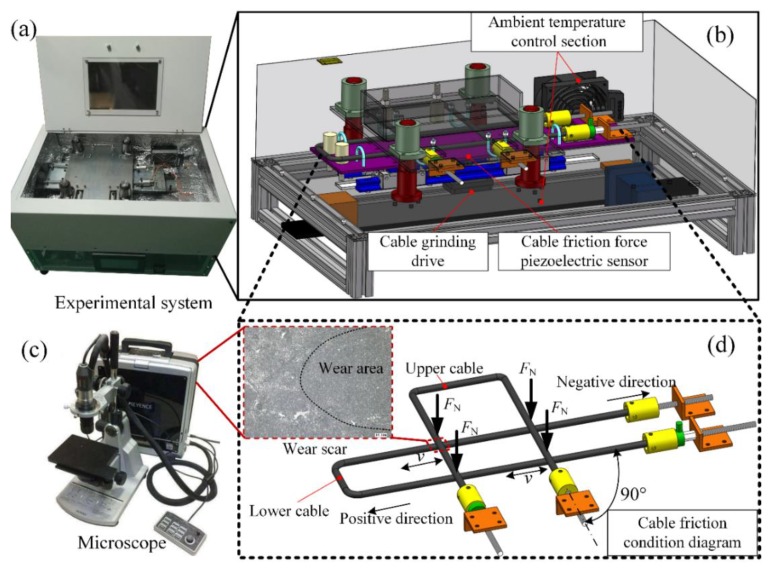
Physical system schematic diagrams of the experimental system, (**a**) friction and wear tester; (**b**) functional model diagram of the friction and wear tester; (**c**) the microscope; (**d**) schematic diagram of the flexible cable friction form.

As shown in Figure 1, the experimental system consisted of a flexible cable friction and wear tester, and a microscope. In Figure 1b, the tester included three main parts: the ambient temperature control section, the cable-grinding drive, and the cable friction force piezoelectric sensor.

As shown in Figure 1a, it is a robot cable friction and wear experiment system designed and built by itself. Its main structure is shown in Figure 1b. The cable-grinding drive component was composed of a servo-motor drive module, a lower cable, and its fixing device. The cable shown in Figure 1b was fixed straight through the hoop, and the connector at the end of the cable is shown in Figure 1d. The cable friction force piezoelectric sensor was set on the servo-motor drive module slider. When the servo motor transmitted the reciprocating motion to the slider, the cable friction force piezoelectric sensor located on both sides of the slider contacted the bracket fixed with the lower cable to generate pressure. When the pressure was equal to the friction force, it was fixed. The lower cable holder moved. The friction force was finally measured by the cable friction force piezoelectric sensor. Referring to the previous research, the intersection angle between the two cables affected the wear—the angle increased with the increase of the wear area [18]. The flexible cables’ wear is similar to the wear between elastomers, which is very different from the rigid wear between hard cables. The depth of flexible cables’ wear causes the most severe damage to their reliability, so the experiment uses a 90° orthogonal form as shown in Figure 1d. The left direction is positive motion and the right direction is negative motion. The friction force is the most intuitive data reflection of friction. In order to research the influence of different working conditions on the friction of flexible cables, the friction force at different working conditions needs to be tested. The surface and chips of wear are the most direct manifestations of friction. It reveals the mechanism of friction to a certain extent. To this end, the microscope (KEYENCE VHX-700F: Keyence (China) Co., Ltd., Shanghai, China.) as shown in Figure 1c is used to observe the cable’s wear surface and wear chips.

### 2.2. Test Management

Among the friction and wear of flexible cables, the orthogonal friction mode is the most severe under the same working conditions (friction frequency, temperature, pressure). The diameter of the selected test cable is 15 mm, it is guaranteed that the friction distance can form a complete friction scar, the reciprocating limit distance is 30 mm. Stay for 0.5 s before the next direction change, which provides a clear boundary division for later friction processing. During the friction test at room temperature (25 °C) and low speed (5 mm/s) by using the above test system, it was found that the appearance of the cable-wear place will change significantly after about 1000 times. So, the friction force recorded by the sampling frequency was 1 kHz and the surface morphology of the wear area significantly changed in the friction area.

The different gradients of those three elements are selected for friction experiments. The flexible cable’s insulation layer (polyvinyl chloride-based polymer) in the high temperature (around 75 °C), the mechanical properties will be changed. Therefore, the maximum level of ambient temperature is chosen to be 70 °C. The experimental parameters and their levels are detailed in Table 1. The working environment of the robot flexible cable is the general factory environment, so the humidity requirement in this experiment is about the general indoor humidity (about 45% RH). Therefore, heating in the form of hot air ensures that the humidity is in a relatively dry state.

The experimental arrangement is generally divided into two parts. In the first part, when the positive pressure is 2 kg, a total of nine groups of friction tests at three relative speeds are performed at three temperatures; in the second part, four different scales of positive pressure are performed in 5 mm/s and 25 °C. As detailed in Table 2, the frictional results of the robot cable under different relative speed, ambient temperature, positive load, and microscopic observation of surface wear were compared and statistically studied.

## 3. Results and Discussions

### 3.1. Mechanical Force Chemical Wear Mechanism

We researched the relationship between the relative speed, ambient temperature, positive pressure, and friction force. The wear mechanism of the flexible cable has been researched by analysis of the surface and the chips of the wear obtained from the wear test.

**Figure 2:**
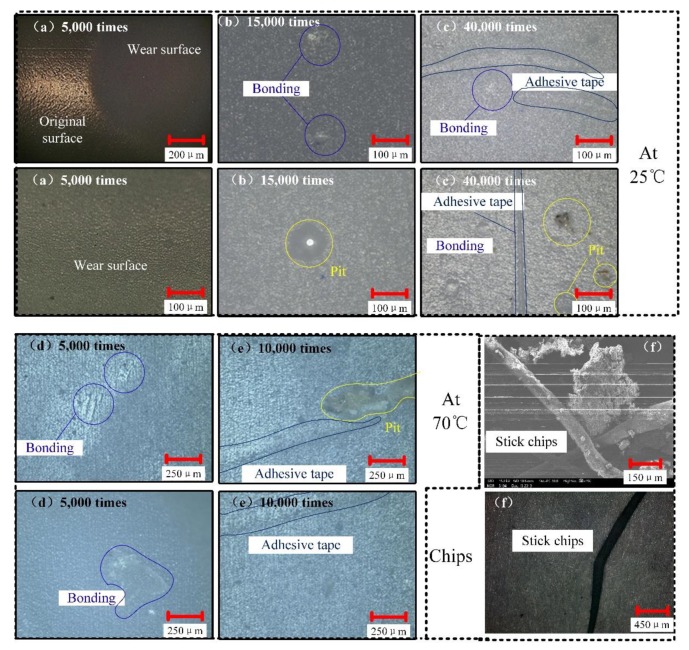
At 25 °C, 2 kg, 5 mm/s, wear surface topography of cable at different friction times (**a**) 5000 times, (**b**) 15,000 times, and (**c**) 40,000 times; at 70 °C, 2 kg, 5 mm/s, wear surface topography of cable at different friction times (**d**) 5000 times, (**e**) 10,000 times, and (**f**) wear chips topography.

The wear surfaces in different stages and the wear chips were observed by the microscope. The observation results were arranged to form a wear surface evolution diagram as shown in Figure 2. Figure 2a–c records wear surface at 25 °C, 2 kg positive pressure, a relative speed of 5 mm/s, and after friction of 5000 times, 15,000 times, and 40,000 times. Figure 2d–e shows the wear surface at 70 °C, 2 kg positive pressure, a relative speed of 5 mm/s, and after friction of 5000 times, and 10,000 times. The chips are shown in Figure 2f, which appeared in the same form of stick shape in different experiments.

It can be seen from Figure 2 that the cable wear phenomenon has been observed. In Figure 2a at 5000 times the wear surface is smoother than the original surface; in Figure 2b at 15,000 times, some bonding points and pit points appeared; in Figure 2c at 40,000 times, a distinct adhesive tape appeared. It can be seen that the bonding of Figure 2d is larger than that of Figure 2e, and when the number is 10,000, significantly, adhesive tapes and pit points can be observed. At 10,000 times, 70 °C, wear marks can be observed similar to those at 40,000 times 25 °C. Under the same conditions, 70 °C, 10,000 times shows the same degree of wear at 25 °C at 40,000 times, so the experiment at 70 °C, 15,000 times is not necessary. Finally, based on the observation of the wear chips like Figure 2f, in the visible chips of the naked cable appears a stick shape. The wear surfaces show that there are no large-scale pits or furrows. We reached the conclusion that the flexible cable-wear pattern of research in this paper is different from those of abrasive wear and fatigue wear.

**Figure 3:**
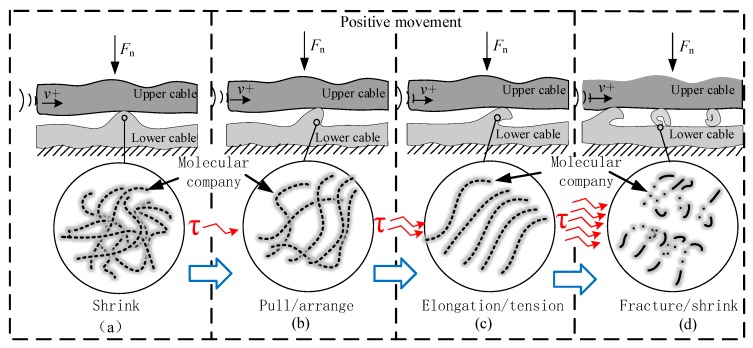
Mechanical force chemical wear principle explanation diagram (**a**) the long chain contraction, (**b**) the long chain stretching, (**c**) the long chains stretch to the limit, (**d**) the long chain break.

Next, we researched the specific mechanism of friction and wear of flexible insulated cables. Based on the friction conditions of the cable and the observation of the wear surface and chips, the friction and wear mechanism of the flexible cable in this research is defined as the mechanical-force chemical [19]. The detailed process is shown in Figure 3a–d.

The last figure shows that: (a) first of all, the two cables in contact form a friction pair. Since the surface layer of the cable is a polymer mainly composed of polyvinyl chloride, the molecular chain of the flocculation mode determines the polymer’s elasticity. As the frictional motion proceeds, the shear stress is generated between the contact surfaces. (b) The contact point on the cable begins to plastically deform along the direction of friction, and the molecular chain begins to stretch, and the distance between the chains becomes wider; as the motion increases, the shear stress increases. (c) The material deformation at the contact point of the cable reaches the limit, and the molecular chain is pulled down to the limit by the action of the large shear stress. (d) When the final stress reaches the yield limit of the cable, the material breaks to form the curl-like wear chips. Its molecular chain breaks to form short-chain free radicals [19].

In the above process, the corresponding mechanical wear and a chemical reaction are generated. When the flexible cable insulation is in the process of reciprocating frictional motion, the shear stress gradually accumulates inside. At the beginning of the friction movement, the polymer constituting the insulating layer, and the shrinking flocculent structure is shown in Figure 3a. With the frictional movement, this flocculent structure begins to be stretched (Figure 3b,c). In that process, the mechanical energy is converted into the internal energy of the polymer, which is used to change the structure of the long-chain polymer. The polymer long-chain molecules are brought into an active state for preparing the subsequent cleavage process. At the same time, this characterizes the onset of the mechanical force chemical reaction of the polymer [20]. Then the polymer long-chain becomes short-chain, and free radicals form of the breaks, and curled wear chips are formed [21].

Those free radicals, because of their high energy, are very likely to undergo chemical reactions with other broken bonds and regenerate polymer long chains. It is most obvious in solid polymers [22,23,24], in which the free radicals have higher activity, and can combine with other short chain molecules to form new long chains at a certain temperature. Therefore, the mechanical force chemical reaction of the polymer has its chemical properties exhibited in a general reversible reaction. This mechanism decides the unique relations between friction force and relative speed, ambient temperature, and positive pressure on the friction movement of flexible cables. In summary, the form of friction between cables is defined as mechanical force chemical friction wear, and the final phenotype of the product of the reaction is friction force and a wear chip.

### 3.2. Effect of Relative Speed on Friction at Different Temperatures

**Figure 4:**
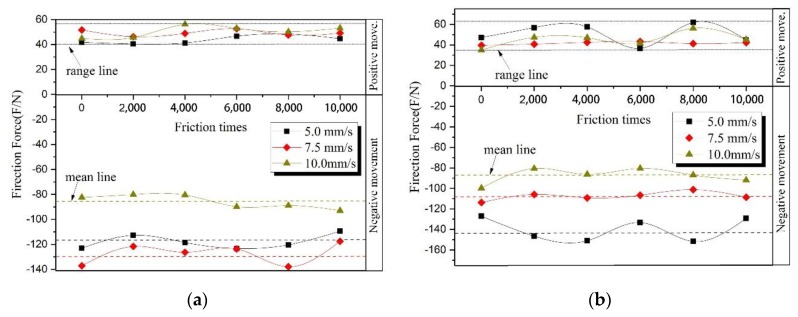

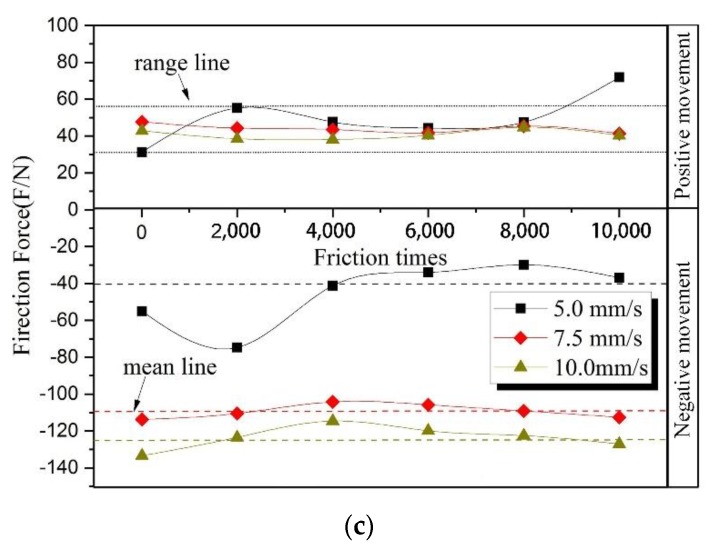
At the different temperature, the relative speed between the cables with the corresponding friction force; (**a**) at 25 °C, (**b**) at 40 °C, (**c**) at 70 °C.

In order to study the effect of the relative speed between cables on the friction, the friction test has been carried out at the three speeds of 5 mm/s, 7.5 mm/s, and 10 mm/s at the same temperature. The friction force results are as shown in Figure 4. We have recorded a completely positive or negative motion as one friction times 2000 on the abscissa axis, representing 2000 positive movements or 2000 negative movements.

As shown in Figure 4, the vertical axis in the figure represents friction force, and the horizontal axis represents the number of friction times. The positive half-axis of the vertical axis corresponds to the force obtained when the relative movement direction of the cable is positive, and the negative half-axis represents the negative direction.

**Figure 5:**
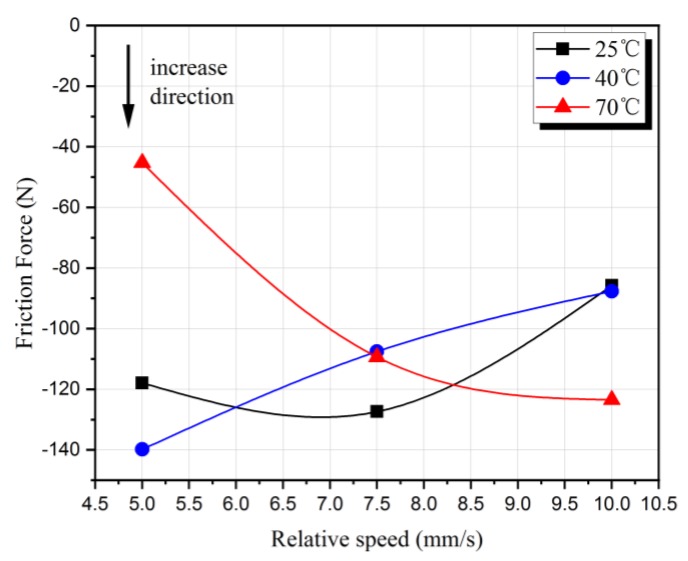
The relationship between the relative speed and friction force in different temperatures.

Figure 4a–c shows friction force and friction times at three different relative speeds at 25 °C, 40 °C, and 70 °C respectively. Due to the greater viscoelasticity of the flexible cable insulation, these results show a more pronounced hysteresis loop than the study of rigid cables by Periera [11] and Wang [25]. It is the reason why the friction force in the positive process is smaller than that in the negative motion process. It shows the correctness and reliability of the study. As shown in Figure 4a–c, in each figure under three different relative speed effects, the difference between the positive friction forces at different speeds is about 20 N, which is much smaller than the 60 N difference between the force obtained from the negative movement. It shows that the negative motion is greatly affected by the speed. So, the friction forces that were obtained from the negative motion mean line have been compared in Figure 3a–c. The friction corresponding to the mean line in each figure is shown as a data point, the relative speed as the horizontal axis, and the friction force as the vertical axis. The relationship between the relative speed and the friction force of the mean value in the negative direction are shown in Figure 5.

As shown in Figure 5, the details of the specific results are as follows: first of all, at 25 °C friction force from small to large 10 mm/s < 5 mm/s < 7.5 mm/s; secondly, at 40 °C friction force from small to large 10 mm/s < 7.5 mm/s < 5 mm/s; finally at 70 °C friction force from small to large 5 mm/s < 7.5 mm/s <10 mm/s. From the above results, it can be seen that the ambient temperature and relative speed together affect the friction of the flexible cable.

Based on the above mechanical force chemical friction and wear mechanisms, it is not difficult to see that the relative speed between the cables directly affects the reaction time, and the energy of the reaction indirectly affects the friction force. Think of a single movement towards the lower cable as a complete mechanochemical reaction. So, the 5 mm/s speed corresponds to the longest overall reaction time, and the 10 mm/s speed corresponds to the shortest reaction time.

Based on the above enactment, the following rules can be obtained:

(1) At 25 °C, due to the low ambient temperature, the reaction requires more energy and the reaction rate is low. The speed of 10 mm/s causes a short reaction time. Although the friction generates a certain amount of heat, it is not enough to greatly increase the reaction rate. The result shows that the friction force is minimal; when the speed is 7.5 mm/s, the heat generated by the friction increases the reaction rate, and the friction is maximized in combination with the slightly extended reaction time; slower speeds of 5 mm/s provide more reaction time, which will generate more friction force than a speed of 10 mm/s. It also shows that the friction speed has less influence on the friction in a lower temperature environment.
(2) At 40 °C, the reaction rate of the mechanical force chemical is improved due to the increase of the ambient temperature. Therefore, a sufficient-force chemical effect reaction can be performed at a speed of 7.5 mm/s, and generate more friction force; and at a relative speed of 10 mm/s, the friction force is still minimal at 40 °C, due to the fact that the speed is too fast to be sufficient for the chemical reaction.
(3) At 70 °C, the time required for the mechanical force chemical effect reaction becomes very short, and the reaction time at the speed of 10 mm/s can perform a sufficient-force chemical effect reaction. At this time, the faster the relative speed, the more heat will accumulate. The more intense the chemical effect, the more the friction force increases with the increase of speed.

### 3.3. Effect of Positive Pressure on Friction

**Figure 6:**
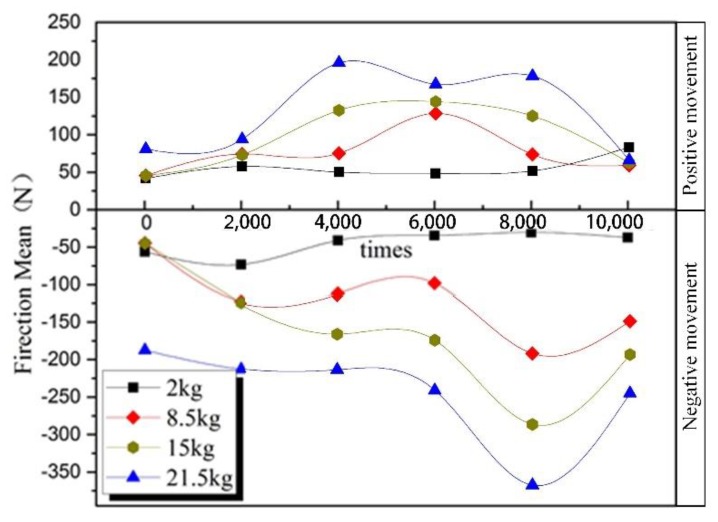
At 70 °C, 5 mm/s, the friction force with different positive pressure.

The friction was greatly influenced by positive pressure. Therefore, the relationship between positive pressure and cable friction was researched in the conditions of 5 mm/s and 25 °C. According to the cable friction condition diagram in Figure 1, the positive pressure was applied. Weights of 2 kg, 8.5 kg, 15 kg, and 21.5 kg were selected as the pressure source. And the test results are shown in Figure 6.

In Figure 6, the four types of friction force curves are analyzed separately. Firstly, the friction force shows increases with the increase of the number of friction times, then decreases slightly, and then increases at about 8000 times. Finally, the friction force decreases. As shown in Figure 6, with the increase of the equivalent value of the forward pressure, the friction force increases to varying degrees, which is reflected in the rapid growth in the early stage, such as the curve corresponding to 8.5 kg, which has increased significantly compared to the curve corresponding to 2 kg. In the friction force curve corresponding to 21.5 kg and 15 kg, it is obvious that the increase of friction force decreases with the increase of pressure. The reason for the above-mentioned change is attributed to the characteristics of the flexible cable forming the friction pair. This is due to the mechanical properties of the flexible cable used in the test and the orthogonal friction form used in the experiment.

**Figure 7:**
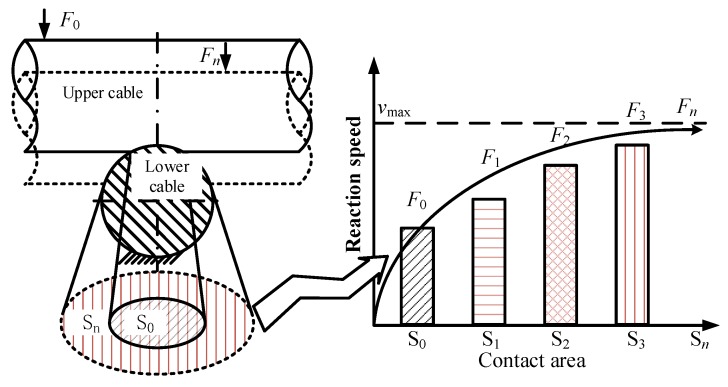
The change of frictional contact area and the corresponding reaction speed.

The reason for this phenomenon is that when the flexible cable is subjected to the positive pressure, its plastic deformation increases quickly, and the frictional contact area increases as shown in Figure 7. The increased contact area will accelerate the mechanical-force chemical reaction, and more polymer long chains will be cleaved, resulting in more friction.

In the orthogonal form of friction, the contact surface formed between the upper and lower cables under the forward pressure of *F*_0_ is *S*_0_ as shown in Figure 7. When the forward pressure increases *F*_n_, the corresponding contact area becomes *S*_n_. As *S* increases, the reaction speed of the mechanochemical reaction gradually increases. The existence of the self-generated deformation limit of the flexible cable makes the contact area have a limited value when the forward pressure increases, and the increase of the contact area with the increase of the positive downward pressure is continuously smaller. Therefore, there is a limited reaction speed for the mechanochemical reaction, and the increase of the reaction speed is continuously smaller, which explains the experimental results of the change of the friction force with the forward pressure as shown in Figure 6.

In addition to the above reasons, the metal conductors inside the cable provide a certain degree of stiffness to the flexible cable, which makes the limit of the flexible cable even smaller. Observed during the friction process, the rod-shaped wear debris as shown in Figure 2f plays a certain role in rolling the friction pair. The greater the forward pressure, the more severely abraded the rod-shaped wear debris, and the stronger the inhibitory effect on friction growth.

### 3.4. Effect of Ambient Temperature on Friction

**Figure 8:**
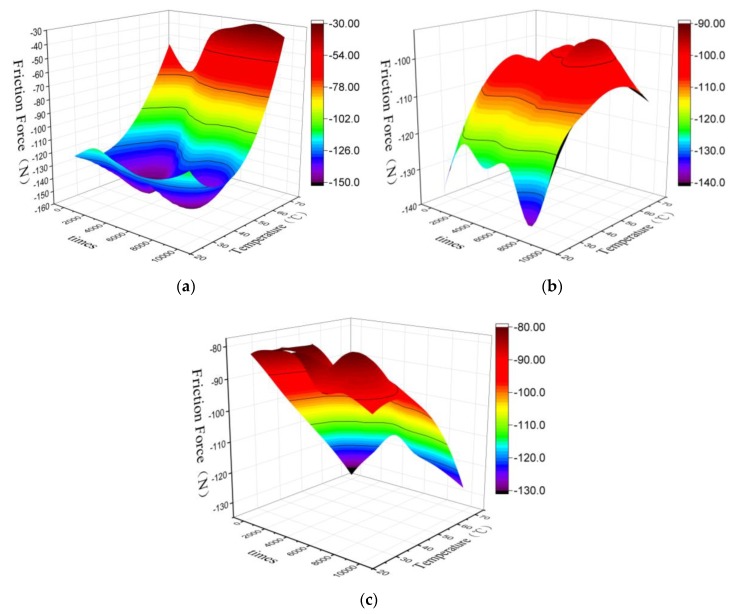
At different relative speeds between cables, the ambient temperature with the corresponding friction force between the cables; (**a**) 5 mm/s, (**b**) 7.5 mm/s, (**c**) 10 mm/s.

The effect of the ambient temperature on the friction force between double cables is studied with the ambient temperature as the main variable. The friction force at three ambient temperatures of 25 °C, 40 °C, and 70 °C are shown in Figure 8. The three-dimensional curved surface is obtained by the fitting of the negative movement friction force. The two coordinate axes formed the horizontal plane in the figure, representing the number of friction times and the ambient temperature.

In Figure 8a at a relative speed of 5 mm/s, along with the increase of temperature, the friction force increases slightly at different friction times and then decreases rapidly under different friction times. And it decreases faster as the ambient temperature increases. In Figure 8b at 7.5 mm/s, as the ambient temperature increases, the friction force decreases gradually under different friction times. After the temperature exceeds 70 °C, there is a small increase in friction. In Figure 8c at 10 mm/s, with the increase of temperature, the friction force increases rapidly under different friction times. And the rate increases with increasing temperature.

**Figure 9:**
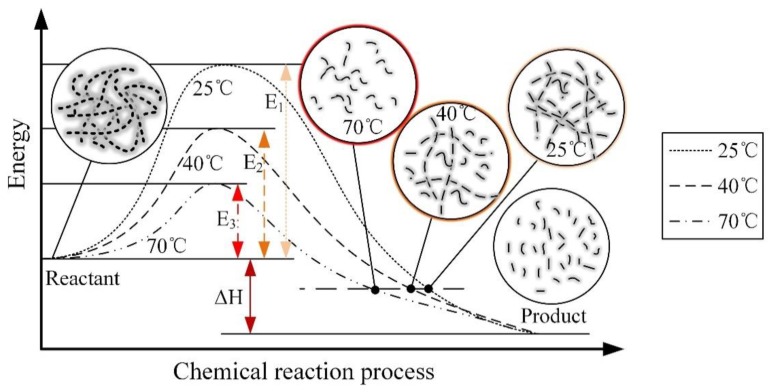
The effect of ambient temperature on mechanical force chemistry.

At different speeds, the relations between ambient temperature and friction force are different. It validates the friction and wear mechanisms described above. The ambient temperature changes the activation energy, which is required for the long chain molecules to reach the reaction state during the mechanical force chemical reaction process. The specific impact is shown in Figure 9.

As shown in Figure 9, the different ambient temperatures that cause the energy required for the polymer long chains to reach an activated state are different. The increases in ambient temperature correspond to the decreases in the energy required during the reaction. The result means that under the same reaction energy, the reaction product at 70 °C is much larger than at 40 °C, and the reaction product at 25 °C is the smallest. For the entire mechanochemical reaction process, its forward reaction process is shown in Figure 9. In the initial test state, the polymer exists in the form of flocculant long chains, and its absorption of mechanical energy reaches the activated state under the action of different ambient temperatures, and finally the long chain becomes a cleavage-by-cleavage reaction. In this process, ambient temperature exists as a catalyst. As shown in Figure 9, the higher the ambient temperature, the less energy E it needs to absorb from the outside. At the same energy, the long chain was broken early in the positive reaction, corresponding to 70 °C as short chain, followed by 40 °C, and 25 °C.

The above theory corresponds to the actual working conditions: the main reaction that occurs in the stage where the friction force increases with temperature in the early stage is the reversible cracking reaction of the polymer, which is the mechanical-force chemical reaction discussed in the previous section. As the temperature increases to an excessively high temperature, the excessive energy accumulation causes an inhibitory effect. The high ambient temperature greatly improves the reverse process of the reaction, and inhibits the generation of friction force, then reduces the friction force.

(1) At 5 mm/s, the reaction time is the longest, and as the number of friction times increases, the accumulated energy is the largest, so this inhibition phenomenon is the most obvious. The friction force decreases most rapidly along with the increasing temperature.
(2) The friction force decreases along with the increase of ambient temperature at a speed of 7.5 mm/s. Because its reaction time is shorter than the time at 5 mm/s, as the value of friction times increases, the energy accumulation is slightly less, which causes the effect to be slightly smaller, and the reduction of friction is lower.
(3) At 10 mm/s, the reaction time is the shortest. The energy accumulated in the reaction process under the first 10,000 friction times cannot reach the heat required for the mechanical force chemical effect to suppress the phenomenon, so it mainly reflects the positive reaction phenomenon. The response of the acceleration is increased, and the high friction is gradually increased. It can be predicted that the friction force will gradually decrease with the increase of temperature.

In general, the temperature will accelerate the wear of cables, which can be observed by observing the evolution of wear surfaces. Comparing Figure 2a–e, it is found that at 70 °C only at about 5000 times, pits formed by the adhesion burn marks and peeling behavior observed at 25 °C and 150,000 times can be observed; at 70 °C, about 10,000 times, the adhesive tape of the friction band similar to that observed at 25 °C times at 40,000 was found. This indicates that the increase of ambient temperature will greatly accelerate the evolution of friction and wear.

## 4. Conclusions

We used the flexible cable friction and wear test system; the friction and wear mechanisms of flexible insulated cables were tested at different relative speeds and ambient temperatures; and positive pressures were researched by orthogonal friction. The influence of the above three factors on the cable friction was analyzed. The research results are as follows:

(1) The form of friction and wear between cables is neither commonly considered as abrasive wear, nor contact wear, but is a form of friction and wear dominated by mechanical-force chemical reactions. It has been verified by the research on relations between the friction and different conditions.
(2) The relative speed between cables will greatly affect the cable friction, which will be greatly affected by the ambient temperature. When the ambient temperature is low (25 °C), the friction speed is the main factor, and the friction heat is the auxiliary influencing factor. At 40 °C, because the lower speed has sufficient reaction time, the friction is large. Friction decreases with increasing speed; in high temperature (70 °C), the ambient temperature is sufficient to provide reaction heat. The friction will increase with the increase of speed.
(3) The ambient temperature will greatly affect the cable friction by affecting the activation energy of the mechanical-force chemical reaction. At 5 mm/s, the friction force decreases most rapidly along with the increasing temperature; the friction force decreases along with the increase of ambient temperature at a speed of 7.5 mm/s; at 10 mm/s, the reaction time is the shortest. The response of the acceleration is increased, and the high friction is gradually increased.
(4) The effect of positive pressure on the cable friction force is mainly to increase the reaction efficiency by increasing the contact area. However, the presence of the copper wires inside the cable reduces the deformation and the contact area. The rod-shaped wear chips generated have a certain lubricating effect and reduce the friction coefficient between cables. These curb the increase in friction force.

This article has done some research on the friction and wear of flexible cables for industrial robots, and the selection of its test conditions is based on the work of general industrial robots. There is insufficient research on the friction and wear of flexible cables under different humidity conditions, gas pressure conditions, and oil-immersed lubrication. We look forward to being able to add to this field.

## Figures and Tables

**Table 1:** Experimental parameters and their corresponding levels.

Ambient Temperature (°C)	Relative Speed (mm/s)	Positive Pressure (kg)
25	5	2
50	7.5	8.5
70	10	15
		21.5

**Table 2:** Experimental details arrangement.

	Order	Ambient Temperature (°C)	Relative Speed (mm/s)	Positive Pressure (kg)
Part 1	1	25.0	5.0	2.0
	2	25.0	7.5	2.0
	3	25.0	10.0	2.0
	4	50.0	5.0	2.0
	5	50.0	7.5	2.0
	6	50.0	10.0	2.0
	7	70.0	5.0	2.0
	8	70.0	7.5	2.0
	9	70.0	10.0	2.0
Part 2	10	25.0	5.0	2.0
	11	25.0	5.0	8.5
	12	25.0	5.0	15.0
	13	25.0	5.0	21.5
